# Investigation of Temperature at Al/Glass Fiber-Reinforced Polymer Interfaces When Drilling Composites of Different Stacking Arrangements

**DOI:** 10.3390/polym16192823

**Published:** 2024-10-06

**Authors:** Brahim Salem, Ali Mkaddem, Malek Habak, Yousef Dobah, Makram Elfarhani, Abdessalem Jarraya

**Affiliations:** 1 LA2MP, National School of Engineering of Sfax, University of Sfax, Sfax 3038, Tunisia; salemiset@gmail.com (B.S.); makram.farhani@gmail.com (M.E.); 2 Department of Mechanical and Materials Engineering, FOE, University of Jeddah, Jeddah 21589, Saudi Arabia; ydobah@uj.edu.sa (Y.D.); ajarraya@uj.edu.sa (A.J.); 3 Laboratoire Roberval, Centre de Recherche de Royallieu, Université de Technologie de Compiègne, Rue du Docteur Schweitzer, CS 60319, 60203 Compiègne, France; malek.habak@utc.fr

**Keywords:** drilling, GFRP/Al interface, temperature, stacking arrangement, thrust force, delamination

## Abstract

This attempt covers an investigation of cutting temperature at interfaces of Fiber Metal Laminates (FMLs) made of glass fiber-reinforced polymer (GFRP) stacked with an Al2020 alloy. GFRP/Al/GFRP and Al/GFRP/Al composite stacks are both investigated to highlight the effect of stacking arrangement on thermal behavior within the interfaces. In a first test series, temperature history is recorded within the metal/composite stack interfaces using preinstalled thermocouples. In a second test series, a wireless telemetry system connected to K-type thermocouples implanted adjacent to the cutting edge of the solid carbide drill is used to record temperature evolution at the tool tip. Focus is put on the effects of cutting speed and stacking arrangement on the thrust force, drilling temperature, and delamination. From findings, the temperature histories show high sensitivity to the cutting speed. When cutting Al/GFRP/Al, the peak temperature is found to be much higher than that recorded in GFRP/Al/GFRP and exceeds the glass transition point of the GFRP matrix under critical cutting speeds. However, thrust force obtained at constitutive phases exhibits close magnitude when the stacking arrangement varies, regardless of cutting speed. Damage analysis is also discussed through the delamination factor at different stages of FML thickness.

## 1. Introduction

Fiber Metal Laminates (FMLs) are a composite material family that combines the properties of metals and fiber-reinforced polymers (FRPs), offering advantages such as light weight, high strength, corrosion resistance, and damage tolerance [1]. These properties make FMLs suitable to use in many industries, including aerospace, automotive, marine, and defense.

The assembly of FML components mainly requires many holes to run through the entire material stack for bolting and riveting [2,3]. This necessitates a drilling pit to cut FML layers that have different mechanical properties and cutting nature. Metal layers generally exhibit plastic deformation, thermal softening, and chip formation, while composite layers exhibit brittle cracking of fibers, relatively ductile cracking of the matrix, fibers pulling out, and possible delamination. Naturally, the complexity of drilling FMLs extends to the type of metal and its chemical affinity, to the tool material, and to the type of fibers and matrix, whether thermoset or thermoplastic, which dictates the drilling behavior and the integrity of the produced surfaces.

The cutting speed of drilling influences various aspects of machining performance and workpiece quality. It affects the surface integrity of drilled holes, particularly in composite and metallic stacks, where higher cutting speeds can lead to increased surface roughness and reduced tool life. For instance, in drilling Ti/CFRP/Al stacks, higher cutting speeds resulted in increased surface roughness and reduced the number of holes that could be drilled before tool failure [4]. Additionally, cutting speed impacts the thermal and mechanical loads during drilling, which can affect the structural integrity of materials. High cutting speeds promote temperature rise, potentially causing thermal damage to the matrix material and altering fiber orientations, which may result in cracks and other defects. Conversely, lower cutting speeds can help maintain better surface quality by reducing thermal effects and mechanical stresses [5]. In drilling operations involving hybrid composite stacks, such as GLARE (glass-reinforced laminate), cutting speed influences the cutting forces and surface roughness. Generally, relatively high speed reduces material resistance due to the softening of phases like aluminum and CFRP [6]. However, the effect of cutting speed on surface roughness is often less significant compared to the feed rate, as observed in drilling CFRP/Al stacks, where the feed rate had a more pronounced impact on surface finish than cutting speed [7]. Moreover, when drilling GLARE composites, higher cutting speeds can reduce cutting forces and improve surface finish, although the choice of cutting speed must be balanced with the feed rate to optimize hole quality and minimize defects such as delamination and burr formation [6].

The stacking sequence in drilling FRPs and metal stacks significantly influences the drilling process and the hole quality. Different stack sequences, such as FRP/metal or metal/FRP, present unique challenges due to the varying mechanical properties and thermal conductivities of the materials involved. For instance, the drilling temperature varies depending on the sequence, as the heat generated during drilling is not dissipated uniformly across different materials. When aluminum is placed beneath CFRP, the continuous and high-temperature chips generated during drilling might pass through the CFRP layer, potentially deteriorating the quality of the hole surface [8]. This was also evident through numerical simulation of the change in drill bit temperature going through CFRP/Ti or Ti/CFRP stacks [9]. This leads to thermal damage, particularly in the interface region, due to the discontinuous heat conduction between layers [9,10]. The sequence also affects the evacuation of chips, especially in metal layers like aluminum, where long and flexible chips can increase the thrust force and temperature, potentially degrading the resin matrix in the composite layer [10]. Additionally, the sequence impacts the surface quality of the drilled holes, with different materials exhibiting varying degrees of surface roughness and diameter tolerance issues. Typically, the difference in elastic moduli of composite and metal layers leads to inconsistent hole diameters across the stack [10]. The choice of drilling parameters, such as spindle speed and feed rate, must be optimized according to the stack sequence to minimize defects like delamination and fiber pull-out in the composite layers and to ensure the integrity of the metal layers [4,11]. Furthermore, the use of customized drill geometries helps mitigate some of the adverse effects of stacking sequences by optimizing the cutting conditions for each material layer, thereby improving the overall hole quality and reducing tool wear [11,12].

Overall, understanding the influence of cutting speed and stacking sequence is crucial for optimizing drilling operations in FRP/metal stacks in terms of required thrust force, generated heat, and surface quality. The drilling thrust force is always considered as the cause of delamination by several researchers in FRP materials, and it is believed that there is a ‘critical thrust force’ above which delamination is initiated [13]. Khashaba et al. [14] found that thrust force increases with cutting speed and feed rate and is significantly influenced by the wear of the drill bit. Zitoune et al. [7] explored the effects of drill diameter and feed rate on thrust force in carbon fiber-reinforced polymer (CFRP)/Al stacks, reporting a direct relationship between these parameters and thrust force. Salem et al. [15] also validated their numerical simulations of the thermomechanical damage that occurs during the machining of CFRP composites with experiments. Their study highlighted the complex interplay between thermal effects and mechanical stresses during drilling. Alongside thrust force, drilling temperature monitoring is also crucial in FML drilling. Due to the confined space in the drilling area, researchers have developed several techniques to reliably record the temperatures throughout the duration of the drilling process. Wang et al. [10] developed a rotating measurement system with embedded thermocouples to measure temperature during the drilling process, while Khashaba et al. [16] used thermal infrared cameras and instrumented drills to monitor the temperature during machining. Most observations have reported that the drilling temperature is correlated to the thrust forces and the delamination factor.

While there are many studies that investigate FML stack drilling behavior, this study presents results to bridge the gap in knowledge to identify drilling strategies that enhance surface integrity and ensure the long-term reliability of Al/GFRP-based composite structures. Specially, the influence of cutting speed and stacking arrangement on delamination and temperature sensed at both the drill bit and the stack interfaces during a hole-making process is addressed.

## 2. Materials and Methods

### 2.1. Specimen Preparation

**Figure 1:**
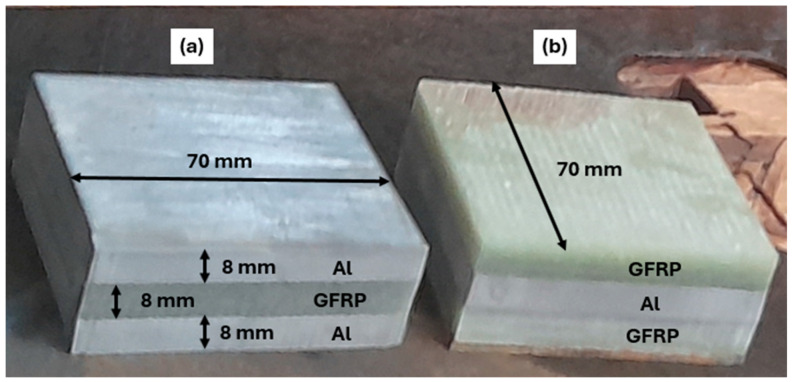
Specimens used in drilling: (**a**) Al/GFRP/Al; (**b**) GFRP/Al/GFRP.

A GFRP composite and aluminum alloy (Al2020) are used to prepare the FML specimens. The GFRP composite used in this work is prepared using the hand lay-up technique. The composite plates are made of ten plies of unidirectional E-Glass cloth of $520\;g.m^{-2}$ belonging to Castro Composites Co., Pontevedra, Spain, and an epoxy resin belonging to Resoltech Co., Rousset, France (reference 1050), mixed manually with 35% of its volume with the hardener 1055S. The adhesion of an Al layer to fiberglass is established by the same mixture made of epoxy 1050 to 1055S resin grade hardener. This resinous mixture minimizes discontinuities in behavior and properties at interfaces between metallic and composite phases, where temperature is sensed and investigated. The assembling is arranged using a clamping system capable of 0.45 MPa [17]. To ensure uniform adhesion, the specimens made of 3 layers are held within the clamping system at room temperature for 24 h. Then, specimens are cut into rectangular plates of 70 mm × 70 mm × 8 mm in dimension. GFRP/Al/GFRP and Al/GFRP/Al composites are specially considered to investigate the effect of stacking arrangement on temperature evolution and localization (Figure 1).

### 2.2. Experimental Setup

**Figure 2:**
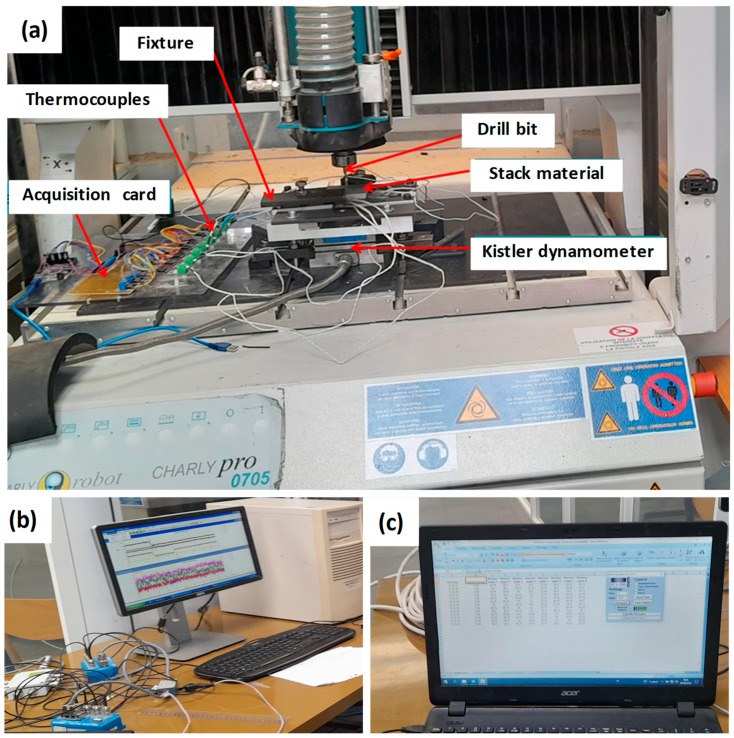
(**a**) Charly robot CPR0705 experimental setup; (**b**) cutting force acquisition system; (**c**) temperature acquisition system.

The experiments are conducted upon two separate Charlyrobot CNC machines (Model CPR0705 and Model CRL1700) supplied by Mécanuméric, Marssac-Sur-Tarn, France. On the first machine, CPR0705, the thrust force and torque are measured by a Kistler dynamometer (model 9257B) connected to an acquisition system through multichannel amplifier 5073A. The results are acquired using Kistler DynoWare EX+ (2825A) software. To ensure a high measurement accuracy of thrust force, the specimen to be drilled is fixed at the center of the dynamometer (Figure 2a).

The drilling tests are performed on the two composite stacks, i.e., GFRP/Al/GFRP and Al/GFRP/Al. Four different cutting speeds of 71, 95, 119, and 142 m min^−1^ and a constant feed rate of 1 mm s^−1^ are used for the design of experiments. All cutting trials are conducted in dry conditions. The drilling process is carried out in all specimens, using a solid carbide drill of 6.3 mm diameter, 5° cutting angle, 150° point angle, 30° Helix angle, and 80 mm working length.

**Figure 3:**
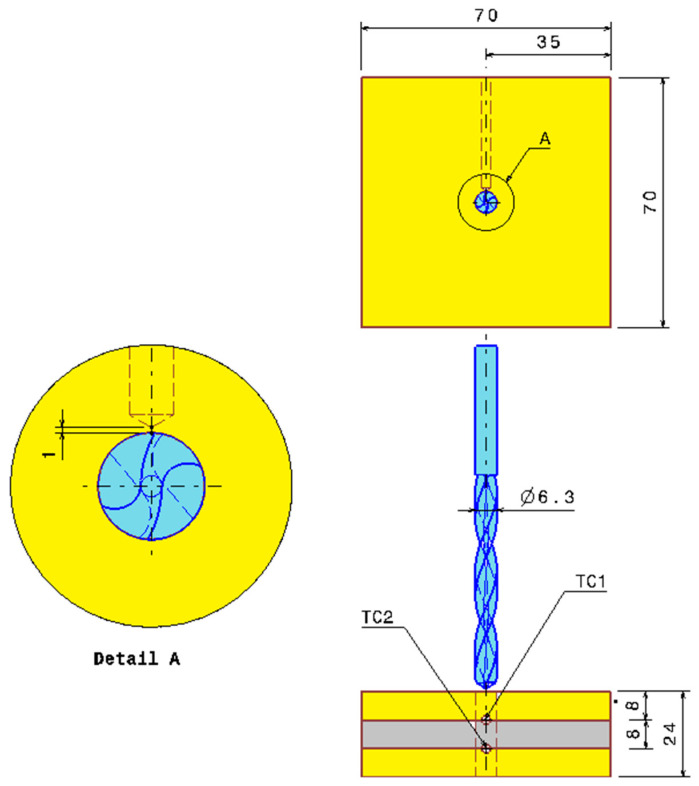
Schematic of positions of installed thermocouples.

On both CNC machines mentioned previously, a measurement system is employed to acquire drilling temperature at the interfaces of the GFRP/Al within the GFRP/Al/GFRP and Al/GFRP/Al configurations. Two K-type thermocouples supplied by Electronic Shop Co., Sfax, Tunisia, with operational range of [−270, 1370] °C are embedded into small holes 2 mm in diameter, at a distance of 1 mm away from the hole wall. Figure 3 shows the side and top views of the drilling configuration, including the drill bit in position relative to the composite stack, and the TCs’ location for temperature sensing. The enlarged view presented as ‘Detail A’ highlights the location of the TCs by referring to the hole wall. The diameter of the thermocouple wire is 0.2 mm, thin enough to avoid disturbing the temperature field in the interfaces of stacks. The thermocouples (TCs) are interfaced with an Arduino Mega board, which is connected to a computer via a USB link (Figure 3). The data acquisition process is conducted using PLX-DAQ software communicating with an Arduino application responsible for signal acquisition and data transfer. The temperature measurement system operates with a sampling frequency of 4 Hz.

**Figure 4:**
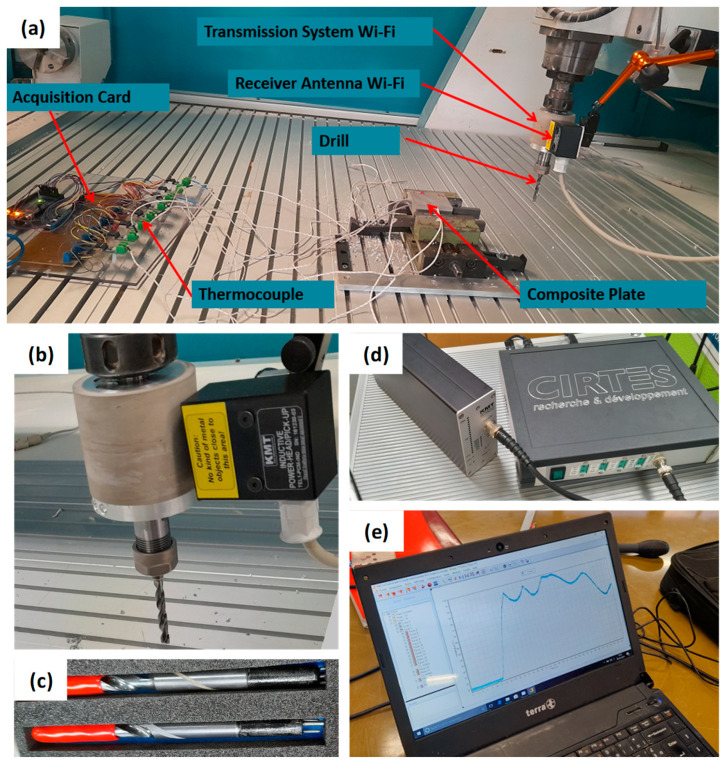
(**a**) Experimental setup Charlyrobot CPR0700; (**b**,**c**) tool equipped with thermocouples; (**d**,**e**) tool temperature acquisition system.

On the second machine, CRL1700, a direct measurement of the tool temperature during drilling operations is conducted using a wireless telemetry system connected to a K-type thermocouple, implanted adjacent to the cutting edge (0.5 mm) of the solid carbide drill. ACTARUS developed, calibrated, and validated the proposed system. The signal captured by the thermocouple embedded in the tool is sent to the tool holder, then relayed to an amplifier through an integrated transmitting device within the tool holder. A receiver antenna module positioned nearby the tool holder enables rapid data acquisition. Figure 4 illustrates pictures of the tool holder, receiver antenna module, and data acquisition system.

### 2.3. Measurement of Delamination Factor 

**Figure 5:**
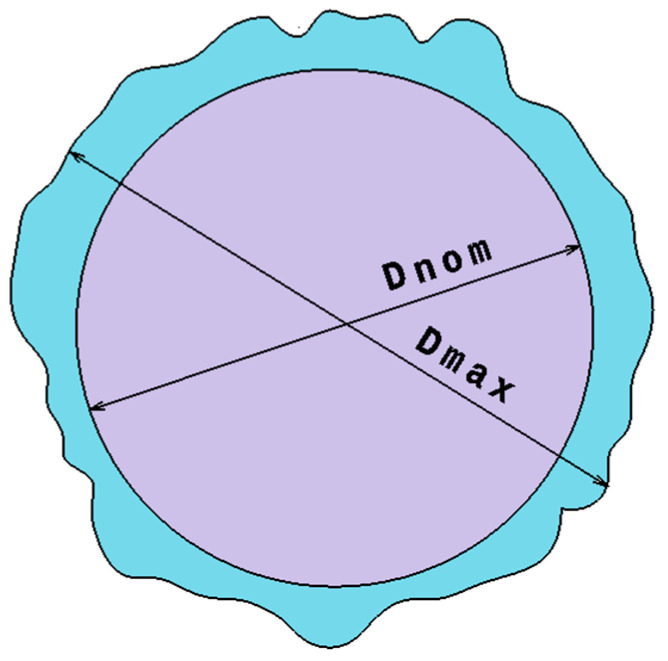
Scheme for measuring the delamination factor.

The drilling-induced damage measured at each hole entry (peel-up) and hole exit (push-out), describes the delamination factor ($F_{d}$) [18]. This factor is defined as the ratio of the maximum diameter of the delaminated area to the nominal diameter of the hole, as illustrated in Figure 5. In the present study, images of every GFRP specimen are taken by a Leica S8 APO stereomicroscope (Leica Microsystems, Wetzlar, Germany) connected to LAS EZ 3.4 software. The measurement of the maximum and nominal diameters in a delaminated hole is assured using VISILOG 7.0 acquisition software. The delamination factor on both hole entry and hole exit is estimated over all GFRP layers based on Equation (1):

(1) $$F_{d}=\frac{D_{max}}{D_{nom}}$$

where $F_{d}$ is a dimensionless measure of delamination state, $D_{max}$ is the maximum diameter overlapping the furthest damaged region, and $D_{nom}$ is the nominal diameter specified by the design.

## 3. Results and Discussion

### 3.1. Drilling Forces

#### 3.1.1. Thrust Force vs. Cutting Stages

**Figure 6:**
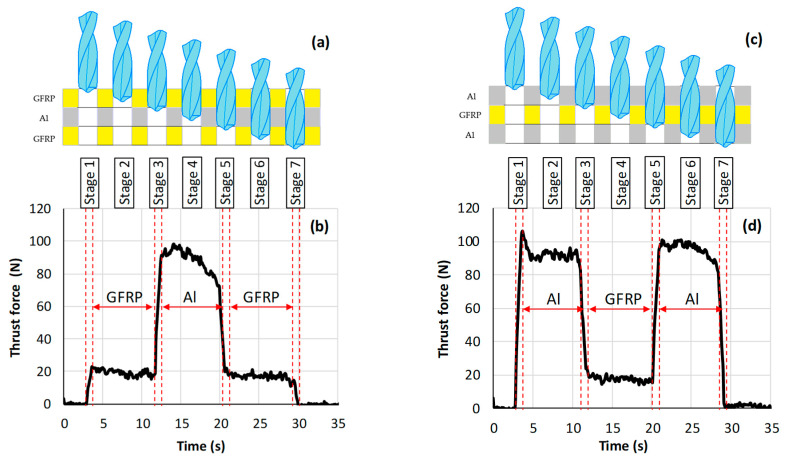
Thrust force recorded when drilling composite stacks at $V_{c}=142\;m\;{min}^{-1}$ and $f=1\;mm\;s^{-1}$. (**a**,**b**) GFRP/Al/GFRP stack and (**c**,**d**) Al/GFRP/Al stack.

In drilling processes, thrust force is of great importance for evaluating the machining performance of a work material. Figure 6a,c illustrate the different cutting stages that should be distinguished for the analysis of the outputs. However, Figure 6b,d show the thrust force plots obtained during different stages of drilling of both the GFRP/Al/GFRP and Al/GFRP/Al stacks, respectively. The plots are recorded for cutting speed of 142 m min^−1^ and a feed rate of 1 mm s^−1^. 

It can be pointed out from Figure 6a that when drilling GFRP/Al/GFRP, the thrust force presents seven stages. Stage 1 indicates that the drilling tool begins to attack the top GFRP plate, and the thrust force sharply increases. In stage 2, the drill tip fully enters the top GFRP phase. Within this stage, the thrust force looks stable. In stages 1–2, the Al plate is not drilled and acts as a support medium for the top GFRP plate.

During stage 3, the drill tip penetrates the top interface zone (GFRP→Al); the thrust force increases substantially in a linear manner until the drill goes through the top interface. In stage 4, the drill tip fully enters the Al phase. In this stage, the thrust force looks roughly stable.

Due to the relatively high hardness of the aluminum alloy compared to GFRP, the force developed whilst cutting the Al phase exhibited much higher magnitude than that recorded whilst cutting the GFRP phase. In stage 5, the drill tip penetrates the bottom interface (Al→GFRP); the thrust force decreases suddenly in a linear manner until the drill goes through the whole interface. In stage 6, the drill tip fully enters the bottom GFRP phase, and the thrust force stabilizes. In stage 7, the cutting tool gradually breaks away from the workpiece; the force signal gradually decreases to zero. Similar stages but opposite evolution of the thrust force plot is found in case of drilling Al/GFRP/Al stacks (Figure 6b).

#### 3.1.2. Thrust Force vs. Cutting Speed

**Figure 7:**
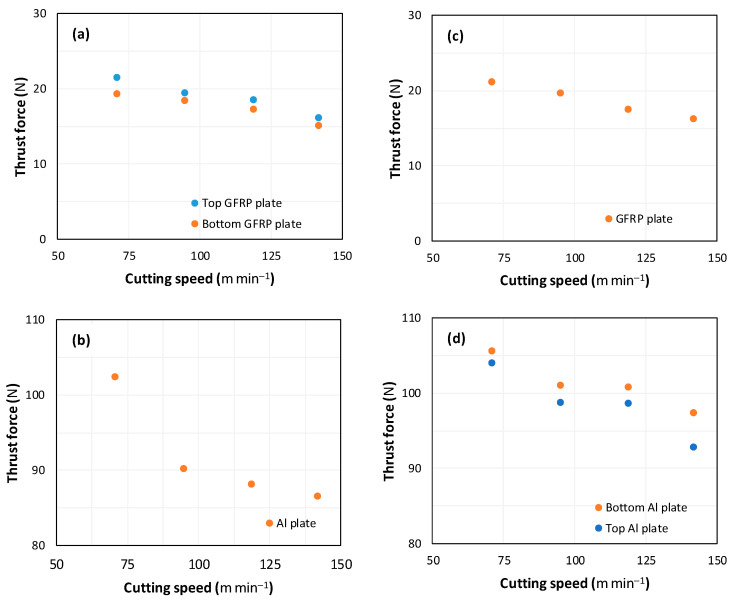
Thrust force vs. cutting speed obtained when drilling hybrid composites at constant feed, i.e., $f=1\;mm\;s^{-1}$. (**a**,**b**) GFRP/Al/GFRP stack; (**c**,**d**) Al/GFRP/Al stack.

Figure 7 illustrates the evolution of thrust force versus cutting speed when drilling GFRP/Al/GFRP and Al/GFRP/Al stack sequences. The findings demonstrate a decrease in thrust force with an increase in cutting speed, which is consistent with previously published studies on drilling glass fiber composites or aluminum [19,20]. 

It can be seen from Figure 7a,b that during the drilling of GFRP/Al/GFRP, the thrust force decreases by 25% for the top GFRP plate, 21% for the bottom GFRP plate, and 15% for the Al plate. The thrust force magnitude measured whilst drilling the Al phase is about five times higher than that obtained for the GFRP phase. When drilling the GFRP/Al/GFRP composite stack at high cutting speed, it can be observed that the thrust force values obtained during drilling of the top GFRP plate are higher than those recorded during drilling of the bottom GFRP plate. This phenomenon is attributed to matrix softening due to the effect of increasing cutting temperature, which increases with cutting speed [7,21].

It can be seen from Figure 7c,d that during the drilling of Al/GFRP/Al, the thrust force decreases by 10% for the top Al plate, 8% for the bottom Al plate, and 22% for GFRP plate. When drilling both sequences of considered composites, cutting speed seems to sensitively affect the evolution of the thrust force in the GFRP phase, while it shows a smaller effect on the recorded values in the metallic phase. When drilling the GFRP/Al/GFRP composite stack at high cutting speed, it can be observed that the thrust force magnitudes for the top Al plate are lower than those for the bottom GFRP plate. The discrepancy observed in thrust force is related to how Al chips are removed during the drilling process. While drilling through the top Al plate, Al chips are efficiently expelled upwards along the drill flutes. In contrast, during the drilling of the bottom Al plate, the metallic chips squeeze and rub against the surfaces of the drilled holes along both the GFRP layers and the Al layer, hence leading to an increase in the thrust force.

### 3.2. Drilling Temperatures

#### 3.2.1. Tool Temperature Analysis

##### Temperature History vs. Stacking Arrangement

It was widely proved that the temperature generated during machining of multi-phase materials plays a crucial role for adjusting the process parameters, particularly the cutting speed [2,3,15,22]. High drilling temperatures lead to heat localization at the subsurface, which alters the mechanical properties of the composite parts close to the hole wall and affects the fresh surface integrity. Thermal effects also promote tool wear, resinous matrix burnout, and damage at both the hole entry and exit, as well as at interfaces. 

**Figure 8:**
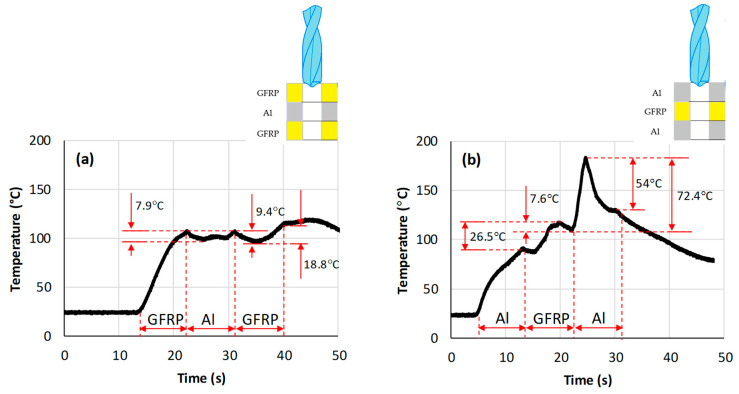
Temperature vs. time obtained when drilling composite stacks at $V_{c}=119\;m\;{min}^{-1}$ and $f=1\;mm\;s^{-1}$. (**a**) GFRP/Al/GFRP stack; (**b**) Al/GFRP/Al stack.

Figure 8 depicts the tool temperature versus stacking arrangement obtained during the drilling of GFRP/Al/GFRP and Al/GFRP/Al stacks. The results are measured at a cutting speed of $119\;m\;{min}^{-1}$ and a feed rate of $1\;mm\;s^{-1}$. From Figure 8a, it can be outlined that when the drill engages the top surface of the GFRP layer, the temperature rises rapidly with the drill advancement. When the drill edges are fully engaged in the GFRP phase, the drilling temperature increases continuously to reach its peak value of 107 °C close to the GFRP/Al interface due to heat accumulation. Then, the temperature decreases slightly through the Al layer within an interval of approximately 7.9 °C. On the one hand, this confirms that the GFRP phase opposes the high dissipation of heat produced by chip removal, compared to the Al layer, which has relatively greater thermal conductivity. In this case of stacking, surrounding GFRP plates act as thermal walls to trap heat generated in the intermediate metallic phase. On the other hand, this yields drastic changes in thermal production because the material removal process switches from brittle fracture mode to plastic deformation mode dominating GFRP chip formation. These observations fit with the findings reported by Wang et al. [10].

The temperature reaches its peak of 116.4 °C at the hole exit, which is only 8% higher than the temperature value measured at bottom interface. When the drill begins to penetrate the bottom layer of GFRP, the drilling temperature decreases by approximately 9.4 °C and then increases by about 18.8 °C to reach its peak value. It can be pointed out that the critical value exceeds the glass transition point $T_{g}$ of the matrix ($T_{g}\approx 110$ °C). This causes thermomechanical damage to the epoxy resins and, hence, affects the integrity of the bottom GFRP phase, while the temperature at the top GFRP layer remains 3.9% lower than the transition point $T_{g}$.

When drilling the top aluminum layer (Figure 8b), the temperature rises at an average rate ($\sim 6.9\;{}^{\circ}C\;s^{-1}$) to reach 91 °C at the interface. Once the drill tip engages the intermediate GFRP layer, the temperature continues increasing by about 26.5 °C until reaching its local peak value (119.7 °C) within the GFRP phase, hence exceeding the $T_{g}$ point, before dropping by approximately 7.6 °C when approaching the bottom interface. When the drill penetrates the bottom metallic layer, the drilling temperature increases drastically with a rate approximating $29.5\;{}^{\circ}C\;s^{-1}$, much higher than the rate recorded at the start of the drilling stage. The absolute peak value is recorded with an increment of about 72.4 °C, after which the temperature falls by approximately 54 °C by referring to the value measured within the bottom interface. 

Figure 8a shows a slight elevation of temperature after the tool exits from the GFRP layer. It is worthwhile to note that once the tool achieves the drilling step, it returns back to its up position and passes back through the three composite layers. The temperature measured after an effective drilling step is, (i) at first, attributed to potential friction generated at the tool/hole wall or tool/chip interface during tool repositioning, which should favor temperature elevation. (ii) Secondly, it can be explained by the critical role that surrounding GFRP layers play in opposing heat loss by trapping it within the tool tip while it returns because of their relatively low thermal conductivity ($\lambda_{GFRP}=2.38\;W\;m^{-1}\;k^{-1}$ [23]). In contrast, Figure 8b shows a smooth and continuous drop in temperature as the tool returns since the surrounding layers are made of Al alloy with a thermal conductivity ($\lambda_{Al}=120\;W\;m^{-1}\;k^{-1}$ [24]) that is high enough to promote heat loss. 

##### Tool Temperature vs. Cutting Speed

**Figure 9:**
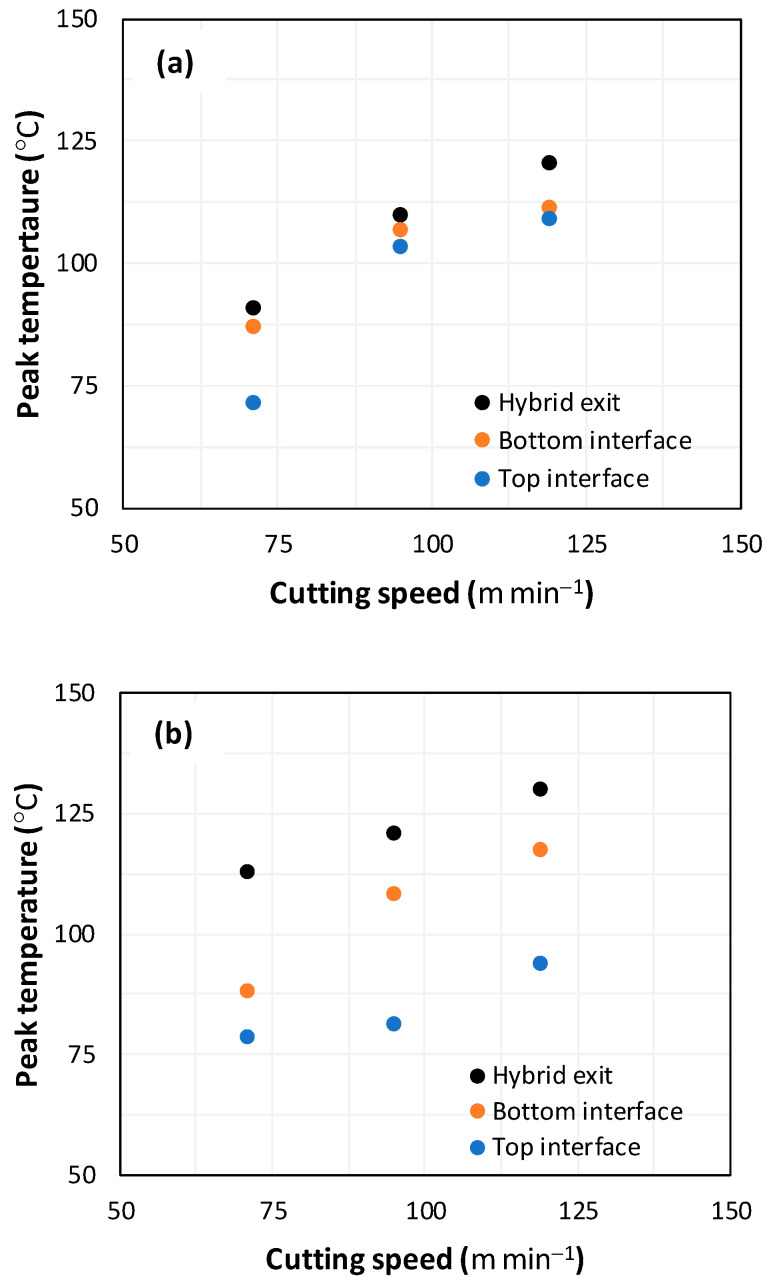
Peak temperature vs. cutting speed recorded at the tool tip when drilling composite stacks at constant feed, $f=1\;mm\;s^{-1}$. (**a**) GFRP/Al/GFRP stack; (**b**) Al/GFRP/Al stack.

Figure 9 illustrates the variation in cutting temperature at the tool/workpiece interface during the drilling of hybrid composites with different stacking arrangements, namely GFRP/Al/GFRP and Al/GFRP/Al. The peak temperature recorded at the top interface, bottom interface, and hole exit is presented in the following figure versus cutting speed. 

Figure 9a proves that cutting speed favors heat generation as can be implicitly observed through the monotonous temperature plots obtained during the drilling of GFRP/Al/GFRP stacks. When the cutting speed increases from 71 to 119 m min^−1^, the temperature within the tool tip increases by 53% when it engages the top interface, 27% when it engages the bottom interface, and 32% when it exits the stack. Rationally, the heat accumulates while the tool advances through the composite layers due to the friction mechanisms acting sensitively in correlation with the cutting speed, at the fresh surface [3]. 

However, Figure 9b shows the evolution of cutting temperature versus cutting speed when drilling the Al/GFRP/Al stack. From measurements, the temperature at the tool tip exhibits quasi-linear evolutions with cutting speed. It attains an increment of about 19% when the tool penetrates the top interface, 33% when it reaches the bottom interface, and 15% when it leaves the hybrid, while the cutting speed varies from 71 to 119 m min^−1^. From Figure 9a,b, the temperature values at both the top and bottom interfaces of GFRP/Al/GFRP are higher compared to those obtained at Al/GFRP/Al interfaces. This discrepancy fundamentally results from the heat accumulation developed because of the net difference in thermal conductivities of constitutive layers ($\frac{\lambda_{Al}}{\lambda_{GFRP}}\approx 50$).

#### 3.2.2. Workpiece Temperature

**Figure 10:**
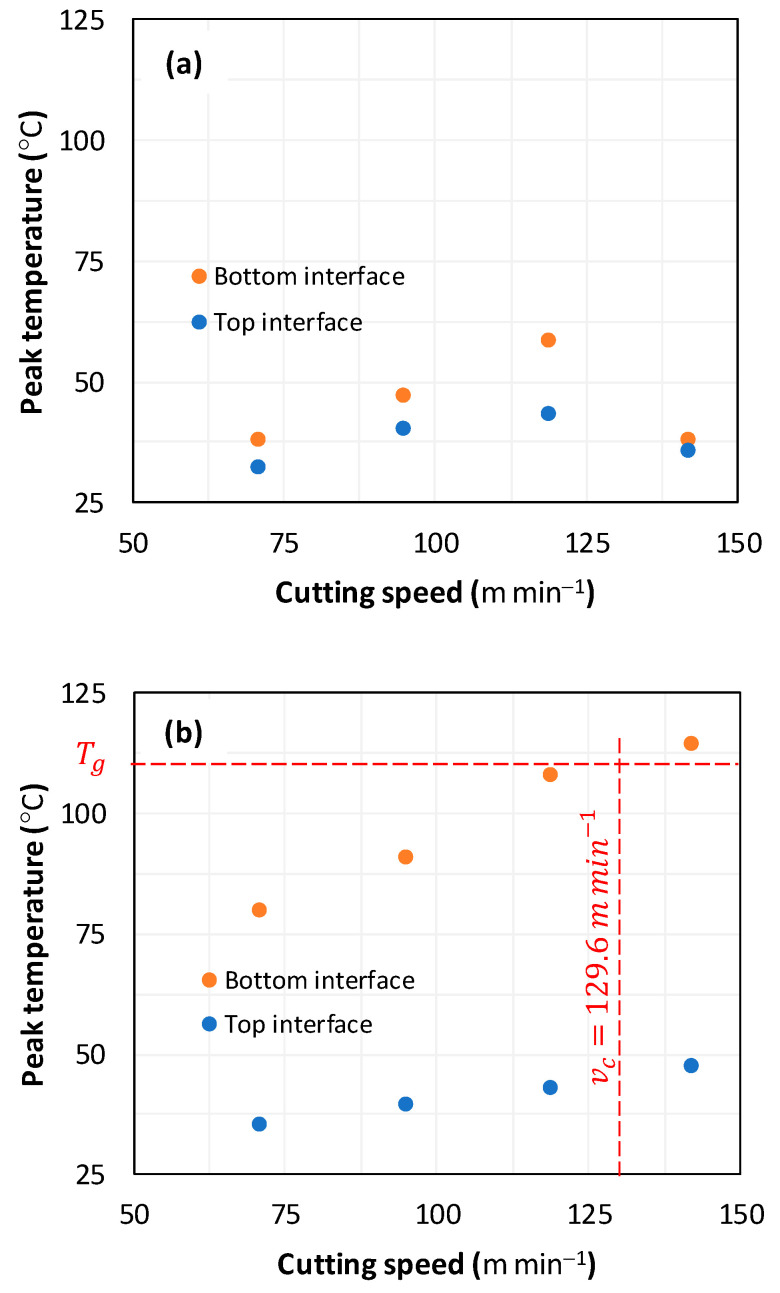
Peak temperature vs. cutting speed obtained at the top and bottom stacking interfaces when drilling composite at constant feed, $f=1\;mm\;s^{-1}$. (**a**) GFRP/Al/GFRP stack; (**b**) Al/GFRP/Al stack.

Figure 10 illustrates the peak temperatures measured using thermocouples preinstalled within both the top and bottom interfaces of considered composite stacks. The TCs are positioned 1 mm away from the hole wall. 

Referring to the plots, the peak temperature increases versus cutting speed except when drilling the GFRP/Al/GFRP composite stack at 142 m min^−1^. The highest peak value is recorded at the bottom interface regardless of the cutting speed and stacking arrangement. Typically, when drilling Al/GFRP/Al composite stacks at the highest cutting speed, the peak temperature at the bottom interface exceeds the glass transition point of the epoxy matrix by a peak-to-transition temperature ratio of $\frac{T_{max}}{T_{g}}=1.04$, which should harm the structural integrity of the fresh surface of the GFRP phase and subsurface as well. However, the most critical ratio obtained at the bottom interface when drilling GFRP/Al/GFRP remains significantly lower at $\frac{T_{max}}{T_{g}}=0.53,$ which entails more appropriate conditions for cutting FML structures. Assuming that peak temperature varies perfectly linearly with cutting speed, it can be pointed out that the maximum allowable cutting speed might be applied so that the peak temperature does not exceed the transition point of the matrix, ${\left.v_{c-max}\right|}_{T=T_{g}}=129.6\;ms^{-1}$. This allowable speed is the practical value to not exceed for ensuring good integrity of the hole wall when drilling the Al/GFRP/Al stack structure (Figure 10b), especially at the most vulnerable region, i.e., bottom interface.

While the aforementioned cutting conditions induce catastrophic heat in drilling Al/GFRP/Al stacks, they seem to be appropriate and safe enough for drilling GFRP/Al/GFRP since the peak temperature at both the top and bottom interfaces still remains far below the glass transition point. 

The discrepancy in thermal properties between the composite and metallic phases induces observable discontinuities when the tool engages the interfaces under critical cutting parameters, e.g., high cutting speeds, stacking arrangement. It is worth noting that the specific heat ${(c}_{p})$ of GFRP is about $700\;J\;{kg}^{-1}\;K^{-1}$ [23], while it is about $875\;J\;{kg}^{-1}\;K^{-1}$ for aluminum alloy [24]. Thus, when drilling Al/GFRP/Al, the composite phase is subjected to the heat flowing from both the top and bottom Al plates with the highest $c_{p}$ value. Inevitably, this promotes heat to accumulate at the interfaces of constitutive stacks before flowing to the whole GFRP phase, as can be seen from Figure 10b.

### 3.3. Damage Analysis

#### 3.3.1. Delamination Factor vs. Stacking Arrangement

**Figure 11:**
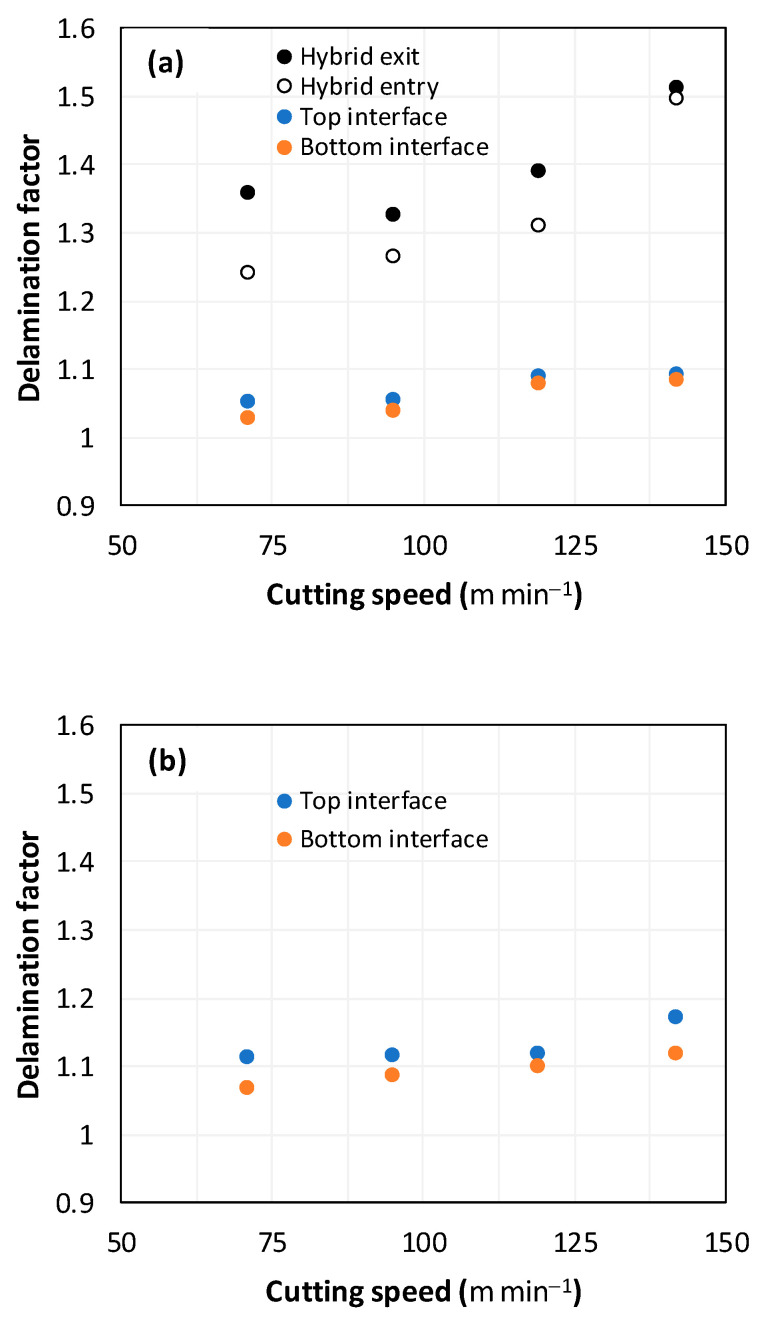
Delamination factor vs. cutting speed obtained when drilling composite stacks at constant feed $f=1\;mm\;s^{-1}$. (**a**) GFRP/Al/GFRP stack; (**b**) Al/GFRP/Al stack.

Assessing delamination during drilling is crucial for enhancing the performance of components made from FML composites. Figure 11 shows the sensitivity of the delamination factor to cutting speed as described in Equation (1).

Figure 11a shows the delamination state measured on hole entry and at the top interface, bottom interface, and hole exit of GFRP plates when drilling the GFRP/Al/GFRP stack. The top and bottom interfaces exhibit less critical delamination factors than that induced at hole entry and hole exit, while $F_{d}$ values obtained on both interfaces look to be close. The intermediate Al plate acts to oppose mechanical damage by supporting the GFRP phases at both interfaces. At hole exit, GFRP undergoes serious delamination since it is free from contact with any part. This behavior is in good agreement with the evolution of drilling temperature, which entails that mechanical and thermal effects act within the composite phase in close interaction. At hole exit, delamination appears more severe than at hole entry since $F_{d}$ reaches critical values referring to an effective diameter about 30 to 50% higher than the expected nominal diameter, i.e., ${1.3\;\times \;D_{nom}\leq D}_{max}\leq 1.5\times D_{nom}$.

However, when drilling the Al/GFRP/Al stack, the delamination factor investigated at the hole edge of the top and bottom sides of the intermediate GFRP plate is found to be slightly higher (Figure 11b) if compared to the values obtained over the interfaces of the GFRP/Al/GFRP stack. This increase in $F_{d}$ is substantially attributed to the deformation of the Al layer occurring due to a rise in the thrust force and the metallic chip flow during the engagement of the tool. Nevertheless, the $F_{d}$ factor exhibits an increasing tendency with the cutting speed similar to that observed in Figure 11a.

#### 3.3.2. Damage Inspections

Table 1 shows the damage morphology at the hole entry, top interface, bottom interface, and hole exit of GFRP during drilling of GFRP/Al/GFRP versus cutting speed. The first column of Table 1 shows the critical damage state at the hole entry, expressed by a relatively wide heat-affected area at the top surface. In the second and third columns of the table, both the top and bottom interfaces exhibit less damage, likely by dint of the support provided by the intermediate Al layer.

The micrographs presented in the last column of Table 1 demonstrate catastrophic burrs owing to uncut fibers at the hole exit. However, this phenomenon attenuates increasingly with increasing cutting speed, which coincides with the decrease already observed in the thrust forces (Figure 7a). Severe thermal damage mechanisms resulting in heat accumulation seem to act together with the mechanical damage mechanisms owing to thrust force, accentuating the delamination state. However, thermal damage is found to be capable of dominating mechanical mechanisms (fiber failure, matrix cracking, interface failure, etc.) during critical temperature rise.

Table 2 shows the damage morphology at both the top and bottom interfaces of the intermediate GFRP phase obtained during drilling of Al/GFRP/Al at different cutting speeds. At relatively low cutting speed, e.g., 71$\;m\;{min}^{-1}$, the top and bottom surfaces of the composite phase exhibit good finish quality. However, more critical damage can be seen surrounding the surface finish at higher cutting speed, where critical fragmentation occurs, as can be typically observed at hole entry obtained at $95\;m\;{min}^{-1}$.

Regardless of the cutting speed, the delamination induced at hybrid interfaces of the two considered stacking arrangements is found at close magnitudes as discussed in Figure 11. At the interface, the Al phase is in direct contact with GFRP and, hence, act to limit delamination progress by preventing local bending, which might be caused by the thrust force due to the advancement of the tool. When the top or bottom surface of the composite phase is free, i.e., GFRP/Al/GFRP, damage is more likely to occur at hole entry and hole exit, as can be seen in Figure 11, where the hybrid exhibits catastrophic failure. Metallic chip flow at relatively elevated temperatures likely leads to more damage, particularly at hole exit. It can be pointed out that chip temperature varies sensitively with tool temperature, which reaches a peak value ($T_{max}=107.2\;{}^{\circ}C$) approximating the glass transition point of the GFRP matrix, which promotes critical thermal damage at the bottom GFRP layer.

## 4. Conclusions

This study deals with the cutting behavior of FML composites made of Al alloy and GFRP stacks using a solid carbide twist drill. Focus is specially placed on the impact of cutting speeds and the arrangement of three alternate constitutive phases on drilling temperature, drilling process, thrust force, and delamination factor. From our findings, the following conclusions can be drawn:

1. The drilling process involves seven distinct thermal stages based on the specific characteristics of the tool–material interaction. The most challenging stages are typically detected at interfaces owing to the sudden transition of the tool from composite to metallic phase or vice versa. In fact, coupled chip separation modes and severe transitions of mechanical and physical responses yield serious thermomechanical discontinuities at interfaces.
2. Irrespective of the stacking arrangement, the thrust force developed in GFRP phase is lower than that recorded in the Al alloy whatever the cutting speed used. However, the thrust force recorded at the top and bottom phases of the GFRP/Al/GFRP stack drops by 25 and 21%, respectively, while it falls by only 8% at the intermediate phase of Al/GFRP/Al when the cutting speed varies from 71 to 142 m min^−1^.
3. The temperature history at the tool tip is measured using a wireless telemetry rotational device. The highest peak values are obtained at hole exit regardless of the stacking arrangement and speed. However, the highest temperature increment is recorded when engaging the top interface of the GFRP/Al/GFRP stack. In fact, peak values increase abnormally by 53% while the cutting speed varies from 71 to 142 m min^−1^.
4. The delamination factor increases with increasing cutting speed. Serious delamination defects occurred at the hole exit of the GFRP/Al/GFRP stack as the composite phase is free from external reactions. Significant burning is detected at the hole entry within the GFRP plate, while uncut fibers owing to relatively low cutting speeds are observed at the hole exit of GFRP/Al/GFRP.

Finally, the thermomechanical behavior of FML structures exhibits high sensitivity to stacking arrangement. From a mechanical point of view, the GFRP/Al/GFRP stack seems to be an inappropriate design solution for drilling because it promotes critical damage at hole entry and exit. However, it exhibits acceptable heat partition between the tool and composite stack. Typically, temperature within interfaces remains far below the glass transition point of the GFRP matrix, which informs us of its good structural integrity at interfaces. 

In contrast, drilling Al/GFRP/Al induces relatively low damage but promotes the temperature to increase sharply within the metallic phase under critical speed values, exceeding the glass transition point within the GFRP matrix, which substantially affects the structural behavior and interface integrity. However, reliably monitoring cutting conditions by adjusting the drilling speed and feed ranges might lead to a safe machining process, ensuring good interface integrity, i.e., relatively low thermal discontinuity at interfaces, and successful finish, i.e., relatively low delamination.

However, several key parameters have not been addressed here that potentially influence the heat balance during drilling. Typically, the drill bit material (coated, uncoated) and geometry (diameter, angles, etc.) are expected to sensitively affect the composite stack behavior, particularly at interfaces. This ought to be concretized in a near-future research work.

## Figures and Tables

**Table 1:** Micrographs showing delamination state vs. cutting speed when drilling GFRP/Al/GFRP composite stack at constant feed $f=1\;mm\;s^{-1}$.

Cutting Speed	Hole Entry	Top GFRP Interface	Bottom GFRP Interface	Hole Exit
71 m min^−1^	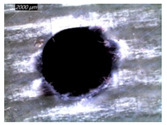	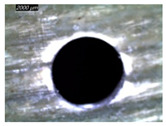	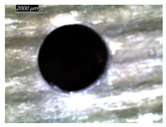	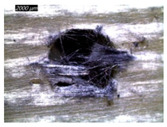
95 m min^−1^	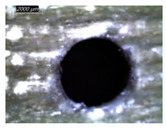	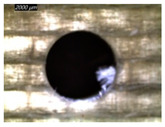	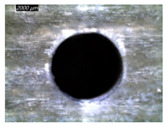	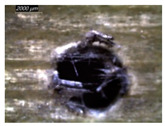
119 m min^−1^	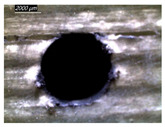	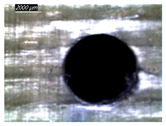	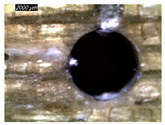	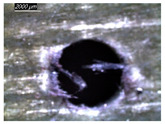
142 m min^−1^	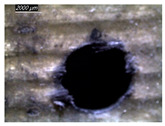	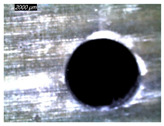	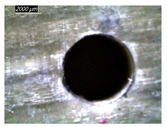	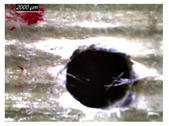

**Table 2:** Micrographs showing delamination state vs. cutting speed when drilling Al/GFRP/Al composite stack at constant feed $f=1\;mm\;s^{-1}$.

	$71\;m\;{min}^{-1}$	$95\;m\;{min}^{-1}$	$119\;m\;{min}^{-1}$	$142\;m\;{min}^{-1}$
**Top GFRP** **interface**	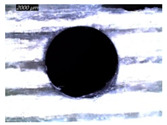	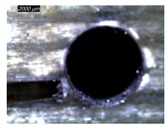	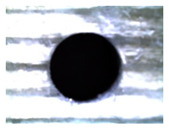	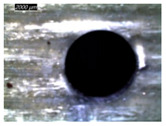
**Bottom GFRP** **interface**	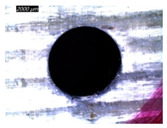	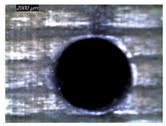	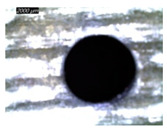	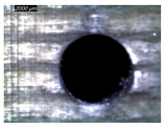

## Data Availability

The original contributions presented in this study are included in the article.

## References

[1] Vlot A., Vogelesang L.B., de Vries T.J. (1999). Towards application of fibre metal laminates in large aircraft. Aircr. Eng. Aerosp. Technol..

[2] Bonhin E.P., David-Müzel S., de Sampaio Alves M.C., Botelho E.C., Ribeiro M.V. (2021). A review of mechanical drilling on fiber metal laminates. J. Compos. Mater..

[3] Dang J., Zou F., Cai X., An Q., Ming W., Chen M. (2020). Experimental investigation on mechanical drilling of a newly developed CFRP/Al co-cured material. Int. J. Adv. Manuf. Technol..

[4] Kuo C.L., Soo S.L., Aspinwall D.K., Thomas W., Bradley S., Pearson D., M’Saoubi R., Leahy W. (2014). The effect of cutting speed and feed rate on hole surface integrity in single-shot drilling of metallic composite stacks. Procedia CIRP.

[5] Brinksmeier E., Fangmann S., Rentsch R. (2011). Drilling of composites and resulting surface integrity. CIRP Ann. Manuf. Technol..

[6] Franz G., Vantomme P., Hassan M.H. (2022). A Review on drilling of multilayer fiber-reinforced polymer composites and aluminum stacks: Optimization of strategies for improving the drilling performance of aerospace assemblies. Fibers.

[7] Zitoune R., Krishnaraj V., Collombet F. (2010). Study of drilling of composite material and aluminum stack. Compos. Struct..

[8] Kim D., Ramulu M. (2005). Study on the drilling of titanium/graphite hybrid composites. Int. Mech. Eng. Congr. Expo. Mater. ASME.

[9] Chen C., Wang A., Zheng Z., Zhao Q., Shi Z., Bao Y. (2023). A study on drilling of CFRP/Ti stacks: Temperature field and thermal damage of the interface region. Materials.

[10] Wang C.Y., Chen Y.H., An Q.L., Cai X.J., Ming W.W., Chen M. (2015). Drilling temperature and hole quality in drilling of CFRP/aluminum stacks using diamond coated drill. Int. J. Precis. Eng. Manuf..

[11] Hassan M.H., Abdullah J., Franz G. (2022). Multi-objective optimization in single-shot drilling of CFRP/Al stacks using customized twist drill. Materials.

[12] Liu L., Qi C., Wu F., Zhang X., Zhu X. (2018). Analysis of thrust force and delamination in drilling GFRP composites with candle stick drills. Int. J. Adv. Manuf. Technol..

[13] Wang H., Wu Y., Zhang Y., Zhang X. (2023). Influence of the temperature-dependent characteristics of cfrp mechanical properties on the critical axial force of drilling delamination. Polymers.

[14] Khashaba U.A., El-Sonbaty I.A., Selmy A.I., Megahed A.A. (2010). Machinability analysis in drilling woven GFR/Epoxy composites: Part II—Effect of drill wear. Compos. Part A Appl. Sci. Manuf..

[15] Salem B., Mkaddem A., Ghazali S., Habak M., Felemban B.F., Jarraya A. (2023). Towards an advanced modeling of hybrid composite cutting: Heat discontinuity at interface region. Polymers.

[16] Khashaba U.A., Abd-Elwahed M.S., Najjar I., Melaibari A., Ahmed K.I., Zitoune R., Eltaher M.A. (2021). Heat-affected zone and mechanical analysis of GFRP composites with different thicknesses in drilling processes. Polymers.

[17] Ostapiuk M., Bienia’s J. (2020). Fracture analysis and shear strength of Aluminum/CFRP and GFRP adhesive joint in fiber metal laminates. Materials.

[18] Joshi S., Rawat K., Balan A.S.S. (2018). A novel approach to predict the delamination factor for dry and cryogenic drilling of CFRP. J. Mater. Process. Technol..

[19] Mahdi A., Turki Y., Habak M., Salem M., Bouaziz Z. (2020). Experimental study of thrust force and surface quality when drilling hybrid stacks. Int. J. Adv. Manuf. Technol..

[20] Liu D.F., Tang Y.J., Cong W.L. (2012). A review of mechanical drilling for composite laminates. Compos. Struct..

[21] Guesmi F., Elfarhani M., Mkaddem A., Ghazali S., Bin Mahfouz A.S., Jarraya A. (2022). Heat analysis of thermal conductive polymer composites: Reference temperature history in pure polymers matrices. Polymers.

[22] Elfarhani M., Guesmi F., Mkaddem A., Ghazali S., Rubaiee S., Abdessalem J. (2022). Thermal aspects in edge trimming of bio-filled GFRP: Influence of fiber orientation and silica sand filler in heat generation. Materials.

[23] Soldani X., Santiuste C., Muñoz-Sánchez A., Miguélez M.H. (2011). Influence of tool geometry and numerical parameters when modeling orthogonal cutting of LFRP composites. Compos. Part-A Appl. Sci. Manuf..

[24] Haddag B., Atlati S., Nouari M., Moufki A. (2016). Dry machining aeronautical aluminum alloy AA2024-T351: Analysis of cutting forces, chip segmentation and built-up edge formation. Metals.

